# Childhood Obesity: A Role for Gut Microbiota?

**DOI:** 10.3390/ijerph120100162

**Published:** 2014-12-23

**Authors:** Marina Sanchez, Shirin Panahi, Angelo Tremblay

**Affiliations:** Department of Kinesiology, Faculty of Medicine, Laval University, Québec, QC G1V 0A6, Canada; E-Mails: marina.sanchez@kin.ulaval.ca (M.S.); shirin.panahi@utoronto.ca (S.P.)

**Keywords:** childhood obesity, gut microbiota, prebiotics, probiotics, body weight, composition

## Abstract

Obesity is a serious public health issue affecting both children and adults. Prevention and management of obesity is proposed to begin in childhood when environmental factors exert a long-term effect on the risk for obesity in adulthood. Thus, identifying modifiable factors may help to reduce this risk. Recent evidence suggests that gut microbiota is involved in the control of body weight, energy homeostasis and inflammation and thus, plays a role in the pathophysiology of obesity. Prebiotics and probiotics are of interest because they have been shown to alter the composition of gut microbiota and to affect food intake and appetite, body weight and composition and metabolic functions through gastrointestinal pathways and modulation of the gut bacterial community. As shown in this review, prebiotics and probiotics have physiologic functions that contribute to changes in the composition of gut microbiota, maintenance of a healthy body weight and control of factors associated with childhood obesity through their effects on mechanisms controlling food intake, fat storage and alterations in gut microbiota.

## 1. Introduction

The prevalence of obesity has increased steadily over the past 25 years, affecting both adults and children worldwide. Approximately 60% of adults and 30% of children in Canada are considered overweight or obese [1]. Obesity is physiologically described as excess body fat resulting from a long-term positive energy balance. This is a major concern as obese children are highly prone to becoming obese adults and are therefore, at high risk of developing severe co-morbidities such as metabolic syndrome, type 2 diabetes and cardiovascular disease [2]. Prevention and management of obesity is proposed to begin in childhood [3]. It is well established that proneness to obesity depends on a complex interplay between numerous factors that are subjected to both genetic and environmental influences. Most of the relevant literature emphasizes that childhood obesity is explained by suboptimal macronutrient composition of the diet and insufficient physical activity; however, recent research has documented the significant impact of more discrete factors such as short sleep duration [4], low dietary calcium intake [5], inadequate feeding behaviors [6] and gut microbiota [7].

Since the human genome has been relatively stable over centuries, it is generally considered that the current obesity epidemic can be primarily attributed to factors associated with a modern lifestyle. Current evidence reveals that even if genetic variation does not appear to be the main determinant of the high prevalence of childhood obesity, there is evidence for a significant role of gene-environment interactions where one’s genetic profile influences the ability to deal with the obesogenic impact of some environmental factors [8].

Current evidence suggests that gut microbiota play a role in metabolic regulation and food digestion and availability [9,10,11]. Gut microbiota is a specific entity within the body which has its own genome whose gene pool is much more abundant than the one of its host. The physiologic functions attributed to gut microbiota have extended to extraintestinal tissues, such as the liver, brain, and adipose tissue, constructing novel connections with obesity [9] and related disorders including type 2 diabetes [12] and cardiovascular disease [10]. Thus, it has the potential to modulate energy regulation as well as systemic inflammation and should be considered as a biological feature that plays a role in the pathophysiology of obesity. Although energy intake may affect the composition of gut microbiota, the extent to which gut microbiota play a causal role in the development of obesity in children and adults is unclear.

A role for the consumption of prebiotics and probiotics and their physiologic functionality in the management of obesity are of interest because studies have reported positive associations between consumption of prebiotics, probiotics and probiotic-containing foods such as dairy and lower body weight [13,14]. Additionally, several strains of bacteria have been tested as a probiotic approach in experimental models of obesity and in human studies demonstrating a decrease in fat mass and body mass index (BMI) [13,15,16,17]. Prebiotics and probiotics are of interest because they have been shown to alter the composition of the gut bacterial community and to affect food intake and appetite, body weight and composition and metabolic functions through gastrointestinal pathways and modulation of gut microflora [18,19,20,21]. 

The following provides a review of the role of gut microbiota in energy balance, differences in gut microbiota between obese and lean individuals, the possible role of prebiotics and probiotics in the regulation of body weight and composition as novel dietary solutions in the prevention and management of childhood obesity and their potential mechanisms of action. 

## 2. Intestinal Microbiota

Intestinal microbiota has been suggested to impact energy balance in animals and humans [11,22] by contributing to energy metabolism from components of the diet and playing a role in how energy is stored and expended [11,23]. Previous research in animals has shown that total body fat was 40% higher in conventionally raised mice compared with germ-free mice even though they had lower food intake [9]. Moreover, Turnbaugh *et al.* showed that transplantation of gut microbiota from *ob/ob* mice to germ-free mice led to a significant increase in total body fat mass compared to germ-free mice that received a gut microbiota transplantation from lean mice [12]. These findings suggest that gut microbiota may play a role in energy harvest and obesity via microbial modulation. 

Human gut microbiota is composed of trillions of bacteria that belong to two predominant bacterial divisions: *Firmicutes* and *Bacteroidetes.* These two phyla are involved in microbial dysbiosis and the development of obesity. Several studies in animals and humans have shown differences in the composition of gut microbiota and energy metabolism between obese and lean populations [24,25]. In a study examining the relationship between the composition of gut microbiota and body fat loss, 12 obese adult men and women randomly assigned to either a low-fat or low-carbohydrate diet for one year showed a lower number of *Bacteroidetes* and higher ratio of *Firmicutes/Bacteroidetes* when compared to lean, normal weight individuals at baseline [24]. However, the ratio returned to normal in those individuals who had successful and sustained weight loss. In another recent study, variations in the fecal microbiota of 12 lean and nine obese individuals during diets that varied in caloric content (2400 kcal/day *vs.* 3400 kcal/day) showed that an altered nutrient load induced rapid changes in the gut bacterial community [26].

The composition of gut microbiota during early life has been suggested to influence development of overweight/obesity in children [27]. In a study examining the impact of perinatal probiotic intervention on the development of overweight and obesity in children over 10 years, 159 women were randomized to either *Lactobacillus rhamnosus* (1 × 10^1^ colony-forming units) and maltodextrin for four weeks before expected delivery and six months postpartum [28]. It was found that early gut microbiota modulation with probiotics may prevent excessive weight gain over the first years of life [28]. This may be one mechanism by which a predisposition for obesity is conferred from the mother to the infant because the mother influences the original inoculums and subsequent development of the infant gut microbiota.

## 3. Potential Dietary Solutions 

The symbiotic cooperation between the gut microbiota and its host could be affected by several factors including dietary habits [29], antibiotics and other environmental factors [30,31,32]. Consumer interest is increasing for foods and food components that may help prevent or treat obesity and related metabolic complications; however, effective dietary countermeasures have not yet been established. Since pharmacological approaches may lead to adverse effects, dietary approaches remain the safest way to reduce obesity and improve metabolic functions, particularly in children. Prebiotics, probiotics and foods containing prebiotics and probiotics are potential tools because of their functional physiological properties. A dysbiosis created by a diet high in fat or low in fibre, for example, is one of the causes of the development of obesity and the increased risk of developing metabolic diseases [33]. Several studies show that these effects on the intestinal microbiota are reversible with improved nutrition [33] and by the administration of prebiotics and probiotics. Consumption of prebiotics and probiotics selectively changes the composition of the gut microbiota in favour of a specific genus and even specific strains in the case of probiotics. 

## 4. Prebiotics

A prebiotic is defined as a “non-digestible fiber or non-digestible food ingredient that beneficially affects the host by selectively stimulating the growth and/or activity of one or a limited number of bacteria in the colon” [34]. The most common prebiotics include inulin and oligosaccharides [35]. It has been shown that prebiotics can have a positive effect on disorders of the digestive system [36,37], immune system [38], hypertension [39], appetite sensations and obesity [20]. It has been suggested that the daily amount consumed in the diet necessary to exert a prebiotic effect is 5–20 g/day [40].

## 5. Probiotics

According to the World Health Organization, a probiotic is a “live microorganism which, when administered in adequate amounts, confers a health beneﬁt on the host” [41]. The two bacterial species that are the most used in probiotic products include *Bifidobacteria* and *Lactobacillus* [42]. It has been shown that probiotics can treat infections or disorders of the digestive system [43,44] and play a role in the prevention of various cancers [45,46] and control of allergy and asthma [47,48]. They also promote positive modulation of the immune system [49], regulation of mood [50] and attenuation of depression [50,51]. Furthermore, probiotics have also been suggested to play a role in the treatment of obesity and related metabolic disorders including hyperglycemia and dyslipidemia [20,52]. Probiotics may be found in foods such as dairy and in supplement form (pills, capsules, tablets and powders). Among the foods in the diet, yogurt was the first to which probiotics were added. Furthermore, it has been demonstrated that some probiotics such as *bifidobacteria* survived better in yogurt than in other matrices including supplements [53].

## 6. Prebiotics, Probiotics and Gut Microbiota

### 6.1. Mechanisms of Action

Human diets may have direct effects on gut microbiota, which ultimately result in changes in the patterns of biochemical reactions in the intestinal lumen. Research supporting the manipulation of the gut microbiota-related pathways by prebiotics and probiotics for treatment of obesity is limited; however, proposed mechanisms include effects on composition and function of the intestinal microbiome. Although the presence of specific bacteria is important, the relative proportions of microbial communities also play a role in energy homeostasis [12].

Microbial imbalances result in an altered intestinal environment that may promote colonic fermentation. Dietary non-digestible carbohydrates can be fermented in the intestinal lumen resulting in production of short-chain fatty acids (SCFA) such as acetate, propionate and butyrate. The profile of SCFAs in the gut reflects the metabolic cooperation between different types of microbiota because no genus of bacteria can hydrolyze all nutrients and none produce these SCFAs upon fermentation. Short-chain fatty acids, which are considered as indirect nutrients produced by the gut microbiota, play a role in energy metabolism and adipose tissue expansion and may act as signaling molecules by stimulating a cascade leading to increased fat storage and energy preservation by binding to G-protein-coupled receptors, GPR41 and GPR43 [54,55]. Studies in *Gpr41*-deficient mice suggested that activation of GPR41 by SCFA are responsible for the release of PYY. Moreover, *Gpr43-*deficient mice fed a high-carbohydrate, high-fat diet had a lower body mass and a higher lean mass compared with wild-type mice [55]. Thus, SCFA produced by fermentation may act as energy substrates and/or metabolic regulators. 

Furthermore, alterations in intestinal bacteria may affect gastrointestinal hormones GLP-1 and PYY which are secreted by endocrine L-cells in response to nutrient stimulus and the orexigenic hormone, ghrelin, which play a role in glycemic control, satiety and energy intake. Prebiotic supplementation has been shown to increase GLP-1 and PYY and decrease ghrelin in humans [56,57] and rodents [58], which in turn inhibit gastric motility via its actions on the ileal brake [59]. Thus, it is possible that prebiotics also delay gastric emptying. 

**Figure 1:**
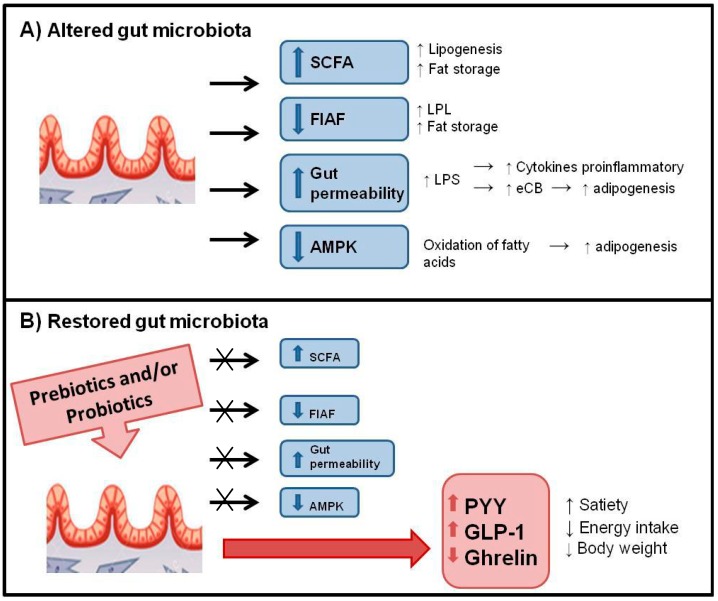
Dysbiosis in the gut microbiota may lead to obesity via different mechanisms. (**A**) An imbalance in intestinal microbiota leads to an increase in SCFA and gut permeability and decrease in FIAF and AMPK; and (**B**) A restored microbiota by prebiotics and/or probiotics may inhibit the mechanisms described in (**A**) and lead to an increase in the hormones PYY and GLP-1 and decrease in ghrelin.

Dysbiosis in the gut microbiota may lead to obesity via different mechanisms (Figure 1). When an imbalance occurs in intestinal microbiota, the bacteria become more effective at extracting energy [60]. The SCFA may act as signaling molecules and stimulate a cascade leading to increased fat storage and energy retention via the GPR41 and GPR43 receptors [54,55]. Microbiota also regulates expression of the FIAF protein (also known as ANGPTL4) which is an inhibitor of lipoprotein lipase (LPL). The modification of microbiota causes a decrease in the expression of FIAF resulting in an increase in LPL activity, a catalyst which captures and stores the fatty acids to adipose and muscle tissue, and increase in lipid storage [9,10]. 

Another mechanism may include the permeability of the intestinal wall. A high-fat diet can alter the composition of the intestinal microbiota and this modification may lead to an increased permeability of the gut barrier by an altered distribution of some tight junction proteins (ZO-1 and occludin). This modification of the wall permeability may also lead to an increase in certain molecules in plasma such as LPS, resulting in metabolic endotoxemia [19,20,61,62,63,64]. The LPS can bind to TLR4/CD14 receptors that are responsible for signaling cascades. These signaling cascades lead to the production of proinflammatory cytokines, particularly TNF-α and IL-6, which are involved in the development of atherosclerosis, obesity and insulin resistance [65]. Several studies have found that chronic infusion of LPS generates a state of insulin resistance [66,67,68]. Furthermore, the change in the composition of gut microbiota stimulates CB_1_ receptors which are responsible for the activation of the endocannabinoid system. This activation is initially responsible for the increased permeability of the intestinal barrier [63]. Thus, LPS molecules can pass through this barrier and cause endotoxemia. By increasing the activation of the peripheral endocannabinoid system, the LPS molecules can also stimulate adipogenesis [11,63]. They also have an inhibitory effect on PPAR molecules which also stimulate adipogenesis [63]. Microbiota may also affect adiposity by decreasing the activity of AMPK in muscle and the liver, causing a decrease in fatty acid oxidation and thus, an increase in adipogenesis [10,69]. 

The modification of gut microbiota also alters secretion of incretins and various gastrointestinal hormones (e.g., GLP-1, PYY, ghrelin) by the cells of the intestinal mucosa. These hormones are involved in glycemic regulation and control of energy intake [70].

### 6.2. Control of Food Intake and Appetite 

It has been suggested that gut microbiota may also affect food intake and satiety via gut peptide signaling [20,71,72]. Gut hormones such as glucagon-like peptide-1 (GLP-1), peptide tyrosine tyrosine (PYY), cholecystokinin (CCK) and ghrelin play a critical role in relaying signals of nutritional and energy status from the gut to the central nervous system in order to control food intake. Experimental studies have shown that GLP-1 is upregulated by prebiotics in obese mice suggesting that alterations in intestinal microflora may stimulate or suppress the secretion of gastrointestinal hormones [58,73,74]. Furthermore, probiotics have been shown to modify the production of satiety hormones when given to rats [75]. 

Clinical studies in adults have shown satiety-inducing effects of prebiotics and probiotics. Although there were no differences in food intake, dairy beverages fermented with *L. acidophilus* and *Proprionibacterium freudenreichii* increased satiety compared with a non-fermented dairy beverage in healthy normal weight female individuals [76]. A study by Cani *et al.* evaluating the effect of consuming prebiotics on gastrointestinal hormone response [57] showed increased gut microbiota fermentation, decreased appetite, improved postprandial glucose responses and higher concentrations of GLP-1 and PYY after two weeks of prebiotic treatment [57] suggesting a role for prebiotics in the modulation of gut hormones. Although there are few studies examining the effect of prebiotics and probiotics on hormonal responses in children, one study investigating the effect of consuming *VLS3*, a combination of eight probiotic strains, for four months on non-alcoholic fatty liver disease in 48 children, found a significant increase in GLP-1 concentrations and reduction in BMI compared to placebo [77]. These studies suggest that gut microbiota may promote the development of obesity and that manipulation of gut microbiota using prebiotics and probiotics may alter gut endocrine function. Further studies are required in order to investigate the pathophysiological basis for the association between gut microbiota and energy homeostasis.

### 6.3. Body Weight Regulation and Body Composition

The potential role of gut microbiota in the development of obesity has led to many investigations on the effects of prebiotics and probiotics on weight management. [13]. In a study examining the effect of a formulation of probiotics (*Lactobacillus rhamnosus*) and prebiotics (oligofructose + inulin) and maltodextrin placebo on weight loss over 24 weeks in 126 obese individuals, it was found that women consuming the formulation lost approximately twice the weight than women consuming the placebo [78]. In addition, the formulation produced a modification of the microbiota by reducing the relative abundance of bacteria of the *Lachnospiraceae* family that are associated with type 2 diabetes [78]. In a randomized, controlled intervention, 87 healthy overweight adults consuming fermented milk containing *Lactobacillus gasseri* SBT2055 (200 g/day) for 12 weeks reduced their visceral and subcutaneous fat, body weight and BMI compared with the control group. Furthermore, consuming two yogurts per day supplemented with *Lactobacillus amylovorus* (10^9^ colony-forming units/yogurt) led to a decrease in total body fat mass [15] suggesting that modulation of gut microbial composition from probiotic consumption may contribute to altered energy metabolism and body composition. The beneficial effect of probiotics on body weight and composition was also observed in a study of 75 obese individuals where the combination of a low-calorie diet and probiotic yogurt significantly reduced BMI and body fat percentage compared with those consuming a low-calorie diet and regular yogurt or no diet and probiotic yogurt over eight weeks [16]. These results suggest that a low-calorie diet combined with probiotic yogurt had a synergistic effect on body composition. Another study examining the effect of a functional yogurt with a mixture of probiotics and prebiotics on metabolic syndrome in 101 healthy adults over 8 weeks found a significant reduction in body weight and BMI in those who consumed the functional yogurt compared with placebo [14]. 

Although there are several studies in adults examining the efficacy of prebiotics and probiotics as potential tools for the prevention and treatment of obesity, few clinical studies have been conducted in children. A study in 70 overweight and obese children examining synbiotic supplementation on cardiometabolic risk factors found that consumption of a combination of probiotic, prebiotic and vitamins A, E, and C over 8 weeks significantly reduced BMI, waist circumference, waist/hip ratio, triacylglycerols and low-density lipoprotein (LDL) cholesterol compared to a placebo [79]. Another study comparing fecal samples from 25 overweight or obese and 24 normal weight children showed lower *Bifidobacterium* and higher *Staphylococus aureus* concentrations in obese children compared with normal weight children [80]. Bervoets *et al.* recently investigated the gut microbiota of 26 overweight/obese and 27 lean children and found that obese children had a higher ratio of *Firmicutes/Bacteroidetes* [7]. Obese children also had higher amounts of *Lactobacillus* spp. and *Staphylococcus* spp. which was positively correlated with plasma inflammatory markers such as C-reactive protein and energy intake, respectively [7]. In another study, fecal samples were analyzed in 15 obese and 15 normal weight children to compare differences in the gut bacterial community [81]. Significantly higher concentrations of the SCFA, butyrate and propionate, and significantly lower concentrations of intermediate metabolites in obese children were observed compared to those with normal weight. The results suggest that the dysbiosis they observed in the obese group may be a factor in the development of obesity [81].

## 7. Conclusions

Prebiotics and probiotics have physiologic functions that contribute to the health of gut microbiota, maintenance of a healthy body weight and control of factors associated with obesity through their effects on mechanisms controlling food intake, body weight and gut microbiota. However, there are a lack of intervention studies examining the effect of prebiotics and probiotics in children in relation to weight management, particularly in the longer-term.

## 8. Future Research

Further studies are needed to elucidate the effects of probiotics and prebiotics on body weight, factors associated with obesity and alteration of gut microbiota in children. Furthermore, the assessment of prebiotics and probiotics in various food matrices including yogurt and dairy products would also provide additional insight into the impact of these components on body weight management in children.
